# Network Pharmacology Analysis and Experimental Study of Yinchen Against Neuroinflammation in Ischemic Stroke

**DOI:** 10.3390/ph18121852

**Published:** 2025-12-04

**Authors:** Minmin Guo, Yijie Ma, Linlin Wang, Ruipeng Ge, You Wang, Gefei Ma, Guanhua Du, Li Li

**Affiliations:** 1 Beijing Key Laboratory of Innovative Drug Discovery and Polymorphic Druggability Research for Cerebrovascular Diseases, Institute of Materia Medica, Chinese Academy of Medical Sciences and Peking Union Medical College, Beijing 100050, China; guominmin@imm.ac.cn (M.G.); mayijie@mail.com.edu.cn (Y.M.); geruipeng@imm.ac.cn (R.G.); wangyou@imm.ac.cn (Y.W.); magefei@imm.ac.cn (G.M.); dugh@imm.ac.cn (G.D.); 2 Xinjiang Key Laboratory of Uygur Medicine, Xinjiang Institute of Materia Medica, Urumqi 830011, China; wanglinlin20@mails.ucas.ac.cn

**Keywords:** Yinchen, *Artemisia scoparia* extract, neuroinflammation, ischemic stroke, network pharmacology, molecular docking

## Abstract

**Objective:** Ischemic stroke (IS) is an acute neurologic injury in which inflammatory responses play a key role. Yinchen, a common medicinal plant used in Traditional Chinese Medicine (TCM), has been proven to possess strong anti-inflammatory effects. However, its efficacy in treating IS remains unclear. In this study, we aimed to investigate the therapeutic potential of Yinchen for IS and the material basis of this potential. **Methods:** The main active components in *Artemisia scoparia* extract (ASE, the extract of Yinchen), were identified by HPLC and MS. The targets of Yinchen and IS were obtained from public databases. Network pharmacology, molecular docking, and experimental investigation were further applied to acquire the core constituents in Yinchen that work against the neuroinflammation that occuring during IS. The neurological outcomes were evaluated in a transient Middle Cerebral Artery Occlusion (tMCAO) rat model. Additionally, the changes in the inflammatory responses in both the ischemic brain and in lipopolysaccharide (LPS)-treated microglial cells were examined using real-time qPCR. **Results:** Four active compounds of ASE, including isochlorogenic acid C (ICGA-C), isochlorogenic acid B (ICGA-B), isochlorogenic acid A (ICGA-A), and chlorogenic acid (CGA), were identified by HPLC and MS. Network pharmacology predicted that 103 compounds of Yinchen had 198 intersection targets with IS. The top five of these targets were TNF, STAT3, IL1B, AKT1, and SRC. Molecular docking results demonstrated that the abovementioned four compounds detected in ASE showed good interaction with all of the above five core targets. Moreover, both the four compounds and ASE were observed to attenuate NO release and suppress the release of various inflammatory factors (TNF-α, IL-1β, IL-6, and MCP-1) in a dose-dependent manner in LPS-induced BV2 microglial cells. ASE was further found to exert neuroprotective effects against ischemia–reperfusion (I/R) injury and inhibit the production of inflammatory factors in tMCAO rats. **Conclusions:** Yinchen exerts an anti-neuroinflammatory effect on IS, and its constituents with high scores binding to five core targets contribute to this effect. This supports its potential as an anti-inflammatory agent for the treatment of IS.

## 1. Introduction

Stroke is a temporary or permanent cerebrovascular occlusion caused by a reduction in local blood supply or local thrombosis, leading to a neurological deficit attributed to an acute focal injury of the central nervous system, which is characterized by high morbidity, a high recurrence rate, and high mortality. It is divided into ischemic stroke (IS) and hemorrhagic stroke. The incidence of IS accounts for 60–70% of all strokes and is one of the leading causes of disability worldwide [1]. Cerebral ischemia can cause neuronal hypoxia, energy depletion, and other problems in a short time, and can ultimately lead to excitotoxicity, calcium overload, apoptosis, inflammation, oxidative stress, and other pathological reactions [2]. Timely recovery of blood flow at the ischemic site has become one of the primary principles in the treatment of stroke, but it also increases the risk of ischemia–reperfusion (I/R) injury due to the generation of harmful free radicals such as the superoxide, hydroxyl, and peroxynitrite radicals [3]. In the United States, recombinant tissue plasminogen activator (r-tPA) remains the sole Food and Drug Administration (FDA)-approved therapy for IS. However, its narrow therapeutic window (less than 4.5 h) and risks such as hemorrhagic transformation significantly limit its use, with only a small percentage of patients benefiting from it [4]. Therefore, from the perspective of pathogenesis, such as inflammation, there exists a great incentive to explore new methods and new drugs for the treatment of IS [5]. In cell-based therapy, the acute phase of IS is marked by inflammation and excitotoxicity, creating a hostile microenvironment that compromises graft survival and integration. As the injury progresses to the subacute phase, inflammation subsides, while blood–brain barrier (BBB) repair and angiogenesis begin, opening a more permissive window for successful graft integration [6]. Thus, suppressing the inflammatory response may help extend this “window of receptivity” following a stroke.

Studies have shown that the degree of inflammatory response in IS is the main factor affecting the prognosis of patients. In the early stage of cerebral ischemia, hypoxia leads to neuronal necrosis and the release of damage-associated molecular patterns (DAMPs), activating microglia and astrocytes and triggering the release of pro-inflammatory cytokines such as tumor necrosis factor-α (TNF-α), interleukin-1β (IL-1β), and other chemokines [7]. These molecules disrupt the blood–brain barrier and promote peripheral immune cell infiltration [8]. TNF-α, along with IL-1β and interleukin-6 (IL-6), is recognized as one of the principal pro-inflammatory cytokines [9]. Following ischemic injury, microglia rapidly initiate localized TNF-α synthesis within the brain parenchyma. This initial response is amplified by subsequent infiltration of peripheral inflammatory cells into the ischemic zone, driving excessive TNF-α production through combined cellular interactions [10]. In addition, one study showed that IL-1β blocking was effective in improving experimental I/R brain injury in mice, and the cerebral anti-inflammatory effect was mediated by a reduction in neutrophilic infiltration and matrix metalloproteinase-2 (MMP-2) tissue levels in the infarcted parenchyma [11]. A clinical study found that baseline inflammatory cytokines, including TNF-α, IL-6, and IL-1β, were independent predictors of late recurrence of IS, suggesting that targeted therapy may benefit high-risk patients with elevated baseline inflammation [12]. For patients who are found to be genetically or biochemically affected by cryptogenic stroke, starting TNF blockade to prevent the development of strokes is essential [13]. Therefore, the search for a novel anti-inflammatory strategy to treat IS is not only beneficial but critical, especially one that has a longer treatment window than current therapies, based mainly on the principle of reperfusion. Recently, Traditional Chinese Medicine (TCM) has attracted more and more attention in the world because of its unique advantages, such as its wide range of sources, potential for systematic treatment, and fewer side effects than other therapies. Thus, in view of the pathogenesis and pathological process of IS, finding effective drugs from TCM has become a focus of current research, which is of great significance for the clinical prevention and treatment of this disease.

The herb Yinchen, which comprises the dried aerial parts of *Artemisia scoparia* Waldst. et Kit. or *Artemisia capillaris* Thunb., is included in the ‘Chinese Pharmacopoeia’. The main chemical compositions of Yinchen include flavonoids, coumarins, chromones, steroids, volatile oil, and phenolic acid, and *Artemisia scoparia* has a wide range of pharmacological activities, such as anticancer, anti-inflammatory, antibacterial, liver-protective, antiatherogenic, antiviral, and neuroprotective functions [14]. In the past, Yinchen was mostly used in clinical practice to protect the liver and gallbladder, with the effects of clearing heat and removing jaundice [15]. But a growing number of studies have shown that *Artemisia scoparia* extract (ASE, the extract of Yinchen) has a certain effect on anti-inflammation and anti-oxidation, which can play a role in inhibiting the production of nitric oxide (NO), prostaglandin E2 (PGE2), TNF-α, IL-6, monocyte chemotactic protein-1 (MCP-1), and reactive oxygen species (ROS) [16]. Zhengan Xifeng Decoction, a TCM prescription written by Zhang Xichun, is composed of *Achyranthes bidentata*, Longgu, *Paeonia lactiflora*, *Scrophularia ningpoensis*, *Asparagus cochinchinensis*, Yinchen, and other TCMs. It is one of the most commonly used prescriptions for the treatment of yin deficiency and yang hyperactivity syndrome in patients with IS. It can reduce the toxic effect of excitatory amino acids and inhibit the expression of endothelin-1 (ET-1) and TNF-α, which play a role in neuroprotection [17]. However, the potential therapeutic effects of Yinchen and its effective active ingredients in the prevention and treatment of IS are still unknown and worth further investigation.

Network pharmacology is a drug discovery approach integrating systems biology, network biology, multi-pharmacology, and other disciplines. It emphasizes that the process of drug action in the body is a complex network, which is a ‘multi-component, multi-target, and multi-channel’ process, and is consistent with the characteristics of TCM. Through the bridge of the intersecting ‘targets’, we can systematically reflect on the complex relationship between TCM components and diseases, explore their mechanisms of action, and analyze the potentially effective components of TCM[18]. Molecular docking technology can quickly and effectively elucidate the pharmacological basis of drugs and improve the accuracy of predictions through computer simulation of the binding modes and affinities between the active components and targets of TCM [19].

Therefore, this study employed a network pharmacology approach to identify key targets and pathways underlying the therapeutic effects of Yinchen against IS and utilized the molecular docking technique to investigate the interactions between the active components identified in Yinchen and Yinchen–IS-associated core targets. Using the lipopolysaccharide (LPS)-induced BV2 microglial cell inflammatory model for in vitro research, the anti-inflammatory activities of these monomeric active compounds and ASE were detected. Furthermore, using a transient Middle Cerebral Artery Occlusion (tMCAO) rat model for in vivo research, the neuroprotective effect of ASE against I/R injury and its anti-inflammatory activity were investigated. Overall, this study is anticipated to provide a theoretical basis for the application of Yinchen in the treatment of IS and contribute to its therapeutic potential as an innovative treatment modality for IS.

## 2. Results

### 2.1. Identification of Bioactive Components from Yinchen

**Figure 1:**
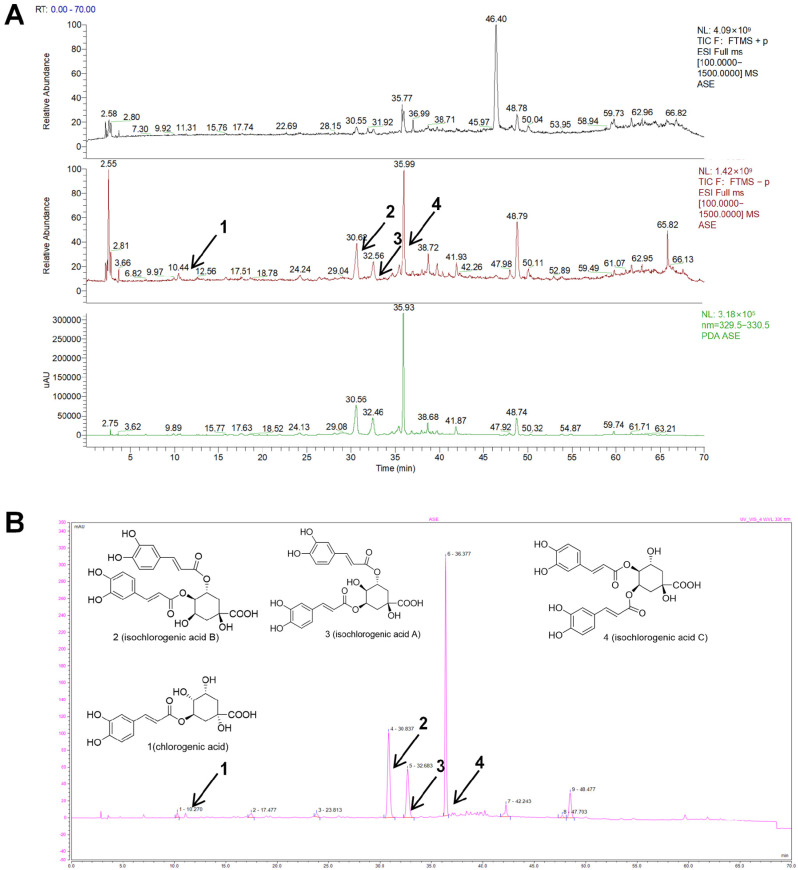
HPLC-HRESIMS and HPLC-UV (diode array detector (DAD)) analyses of ASE. (**A**) (+)/(−) HRESIMS ion current profiles of the ASE and HPLC-UV analysis monitored at 330 nm. (**B**) HPLC-UV (DAD) analysis of the ASE monitored at 330 nm. (1) chlorogenic acid, (2) isochlorogenic acid B, (3) isochlorogenic acid A, and (4) isochlorogenic acid C.

Using High Performance Liquid Chromatography–High Resolution Electrospray Ionization Mass Spectrometry (HPLC-HRESIMS) and High Performance Liquid Chromatography–Ultraviolet (HPLC-UV) analyses, four compounds, including chlorogenic acid (CGA) (1), isochlorogenic acid B (ICGA-B) (2), isochlorogenic acid A (ICGA-A) (3), and isochlorogenic acid C (ICGA-C) (4) were identified in ASE (Figure 1, Table 1 and Table S1). In addition, according to an HPLC analysis and HRMS data, the four characteristic constituents in ASE were identified with their reference standards (Figures S1–S5). Consistent with the results of previous studies, the contents of isochlorogenic acids A, B, and C in ASE were relatively high, while the flavonoid components were extremely low [16]. Therefore, the four compounds identified by the HPLC-HRESIMS and HPLC-UV analyses were the major representative compounds of ASE.

### 2.2. Active Ingredients and Target Screening of Yinchen

To further elucidate the material basis of Yinchen’s potential neuroprotective effects, we conducted a network pharmacological analysis. According to the database queries, 53, 69, and 48 monomeric components were obtained from the TCMSP, TCM Database@Taiwan, and the Chinese Natural Products Chemical Component Database, respectively. After screening and deduplication according to the Lipinski rules and ADMET criteria, 99 active ingredients were finally obtained. Therefore, a total of 103 candidate compounds were collectively identified through the integration of HPLC-HRESIMS/UV analyses (four compounds) with three database searches. The SwissTargetPrediction platform was further used to predict the targets of the active ingredients under the set screening conditions. A total of 4674 targets were obtained, and 829 targets of Yinchen were finally obtained after deleting the duplicates.

### 2.3. Potential Targets of Yinchen in the Treatment of IS

**Figure 2:**
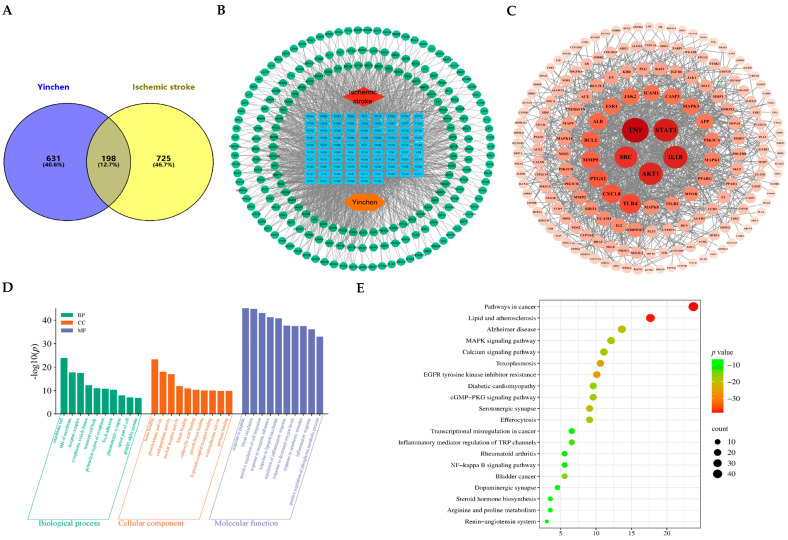
A preliminary screening and network pharmacological analysis of potential therapeutic gene targets of Yinchen against IS. (**A**) A total of 198 common targets of Yinchen and ischemic stroke. (**B**) ‘Drug–active ingredient–intersecting target–disease’ network (**C**) PPI network plotting of the top five intersecting targets by employing Cytoscape software. (**D**) The top 10 most significantly enriched terms in BP, CC, and MF of GO analysis. The Y-axis represents the enrichment count of the target, and the X-axis represents the GO category of the target gene. (**E**) The top 20 pathways with the most significantly enriched targets were selected. The Y-axis represents the main pathway, and the X-axis represents the gene ratio.

The GeneCards and DisGeNET databases were further used to retrieve the disease targets of IS, and 4558 (880 with relevance >4) and 1159 (126 with correlation score ≥0.1) were obtained, respectively. After deleting duplicates, 923 disease targets of IS were finally obtained. The data were uploaded to the Venny2.1 online platform to obtain 198 intersecting targets of Yinchen and IS (Figure 2A).

### 2.4. ‘Drug–Active Ingredient–Intersecting Target–Disease‘ Network

Next, Cytoscape 3.10.1 software was used to construct Yinchen’s ‘drug–component–target–disease’ network. In the network, the red nodes represent the disease, the orange nodes represent the drug, the blue nodes represent the active ingredients in Yinchen, the green nodes represent the intersecting targets, and the edges represent the interactions between the components and the targets. One component often corresponds to multiple targets, and one target may also correspond to multiple components. The network of ‘Yinchen–component–target–IS’ contains 287 nodes and 1720 edges (Figure 2B).

### 2.5. Protein–Protein Interaction Network of Intersecting Targets

To further obtain the core targets of Yinchen against IS, the STRING database and Cytoscape 3.10.1 software were used to perform protein–protein interaction (PPI) analysis on 198 intersection targets of Yinchen and IS. In total, 182 interaction targets and 925 PPI relationships were obtained. The size and color of nodes in the network reflect the closeness of the interactions between proteins. The higher the value obtained, the larger the circle and the darker the color, indicating that the target was more important in the network. The top five intersecting targets were TNF, the signal transducer and activator of transcription 3 (STAT3), IL1B, serine/threonine protein kinase 1 (AKT1), and src proto-oncogene, non-receptor tyrosine kinase (SRC) (Figure 2C). These may be the core targets of Yinchen for the treatment of IS.

### 2.6. GO Functional Enrichment Analysis and KEGG Pathway Enrichment Analysis

In order to clarify the biological functions of the intersecting targets of Yinchen and IS, GO function and KEGG pathway enrichment analysis were performed. The results of GO functional enrichment analysis showed that their biological processes mainly involved circulatory system processes, positive regulation of cell migration, responses to lipopolysaccharides, regulation of inflammatory responses, responses to hypoxia, etc. The cell composition mainly involved membrane rafts, the plasma membrane, the cytoplasmic perinuclear area, dendrites, the cytoplasmic vesicle cavity, etc. The molecular functions were mainly focused on heme binding, protein kinase activity, endopeptidase activity, nuclear receptor activity, G protein-coupled receptor binding, etc. The results of KEGG pathway enrichment analysis showed that the main enriched pathways of key targets were the tumor signaling pathway, the processing of lipids and atherosclerosis, the mitogen-activated protein kinase (MAPK) signaling pathway, the calcium signaling pathway, the cyclic guanosine monophosphate–protein kinase G (cGMP-PKG) signaling pathway, the inflammatory mediator regulation of transient receptor potential (TRP) channels, the nuclear factor kappa B (NF-κB) signaling pathway, and so on (Figure 2D,E). Hence, the results demonstrated the inflammation- and IS-associated core targets of the biological processes of Yinchen in the treatment of IS, further suggesting the role of its anti-inflammatory effects in its potential against IS.

### 2.7. Molecular Docking of Core Components and Targets

**Figure 3:**
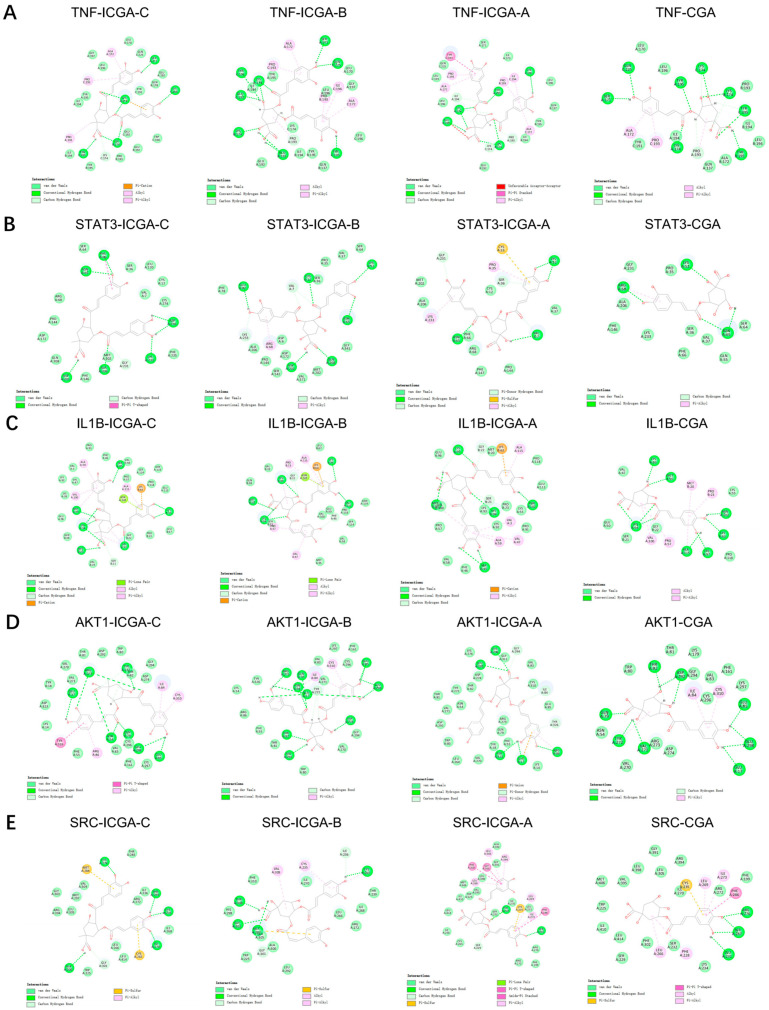
The binding modes of the four compounds (isochlorogenic acid C (ICGA-C), isochlorogenic acid B (ICGA-B), isochlorogenic acid A (ICGA-A), and chlorogenic acid (CGA)) in the ligand-binding sites of (**A**) TNF, (**B**) STAT3, (**C**) IL1B, (**D**) AKT1, and (**E**) SRC.

Next, the four identified ligands of Yinchen, including isochlorogenic acid C, isochlorogenic acid B, isochlorogenic acid A, and chlorogenic acid, were redocked to the five core targets (TNF, STAT3, IL1B, AKT1, and SRC) of Yinchen in the treatment of IS using the CDOCKER program in Discovery Studio 2019. From Table 2, it can be seen that compared with the mean docking scores of the targets and their respective agonists or antagonists, all four compounds showed higher mean binding scores; ranked by their average docking score against the five targets from highest to lowest, the order was isochlorogenic acid C, isochlorogenic acid B, isochlorogenic acid A, and chlorogenic acid. According to the results of molecular docking, the four compounds identified in Yinchen showed good scores in their interactions with all of the above five core inflammatory and IS-associated targets, and it was thus determined that they deserved to be further tested in an in vitro experimental study. The binding modes of the four compounds at the binding sites of the five targets are depicted in Figure 3.

### 2.8. Compounds from Yinchen Decrease the Production of NO in LPS-Induced BV2 Cells

**Figure 4:**
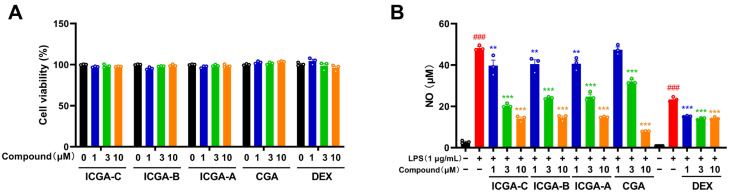
Effects of compounds in Yinchen on cell viability and NO levels in BV2 cells treated with LPS. (**A**) Effects of different concentrations of the four compounds (isochlorogenic acid C (ICGA-C), isochlorogenic acid B (ICGA-B), isochlorogenic acid A (ICGA-A), chlorogenic acid (CGA)) and DEX (1, 3, and 10 μM) on BV2 cell viability. (**B**) The inhibitory effects of the four compounds and DEX at different concentrations (1, 3, and 10 μM) on the NO level of LPS-induced BV2 cells. Data are expressed as mean ± SEM (*n* = 3). ^###^ *p* < 0.001 vs. the Control group; ** *p* < 0.01 and *** *p* < 0.001 vs. the LPS group.

After the above network pharmacology and molecular docking analyses, using LPS-induced BV2 microglial cells as an in vitro inflammatory model, we further investigated the anti-inflammatory effects of these four compounds. Dexamethasone (DEX), a representative anti-inflammatory drug, was used as a positive control. LPS is a potent stimulator of microglial activation and NO production, mimicking the inflammatory conditions observed in IS. Results showed that treatment with the five compounds at concentrations of 1, 3, and 10 μM did not significantly decrease BV2 cell viability (Figure 4A). This indicates that the compounds are non-toxic to BV2 cells at the tested concentrations, suggesting that their potential therapeutic effects are unlikely to be compromised by cytotoxicity within this concentration range. BV2 cells were pretreated with different concentrations of these five compounds (1, 3, and 10 μM) for 24 h. The levels of NO in the cell supernatants were measured using the Griess reagent assay, a standard method for quantifying NO production. Furthermore, the four compounds significantly inhibited the production of NO in LPS-stimulated BV2 microglial cells in a dose-dependent manner. DEX was also found to decrease the NO release of LPS-induced BV2 cells starting at a concentration of 1 μM. But the four compounds exhibited greater inhibitory effects on the production of NO at a concentration of 10 μM than DEX (positive control) (Figure 4B). The results give a preliminary verification of the compounds’ anti-inflammatory effects in vitro.

### 2.9. Compounds in Yinchen Attenuate the Release of Pro-Inflammatory Cytokines in LPS-Induced BV2 Cells

**Figure 5:**
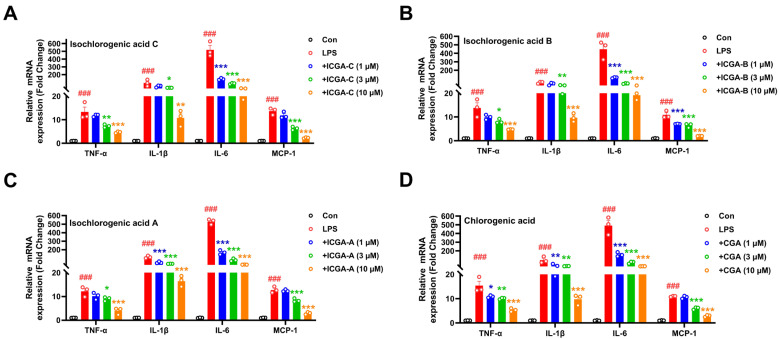
The effects of the four compounds (isochlorogenic acid C (ICGA-C), isochlorogenic acid B (ICGA-B), isochlorogenic acid A (ICGA-A), and chlorogenic acid (CGA)) on the mRNA expression of the pro-inflammatory factors in LPS-induced BV2 cells. (**A**) ICGA-C, (**B**) ICGA-B, (**C**) ICGA-A, (**D**) CGA. Data are expressed as mean ± SEM (*n* = 3). ^###^ *p* < 0.001 vs. the control group; * *p* < 0.05, ** *p* < 0.01 and *** *p* < 0.001 vs. the LPS group.

After confirming that the four compounds from Yinchen showing high scores with all the above five core targets had the effect of reducing NO production, we further investigated whether the compounds had the ability to inhibit the release of pro-inflammatory factors, including TNF-α, IL-1β, IL-6, and MCP-1. Through real-time quantitative reverse transcription PCR (RT-qPCR), it was found that all four compounds had the effect of inhibiting the release of pro-inflammatory factors, compared with the LPS group. Also, these pro-inflammatory factors’ down-regulation was observed to behave in a dose-dependent (1, 3, and 10 μM) manner (Figure 5).

### 2.10. ASE Prevents NO Release in LPS-Induced Microglial BV2 Cells

**Figure 6:**
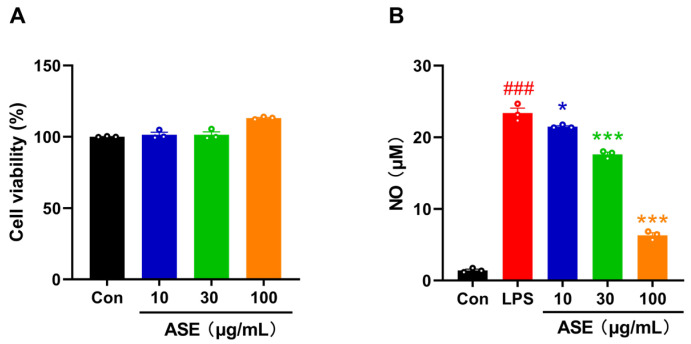
Effects of ASE on cell viability and NO levels in BV2 cells treated with LPS. (**A**) Effects of different concentrations of ASE on BV2 cell viability. (**B**) The inhibitory effect of different concentrations of ASE on the NO levels in LPS-induced BV2 cells. Data are expressed as mean ± SEM (*n* = 3). ***^###^*** *p* < 0.001 vs. the Control group; * *p* < 0.05 and *** *p* < 0.001 vs. the LPS group.

After verifying the anti-inflammatory effects of monomeric active compounds from Yinchen in vitro, we further evaluated the anti-inflammatory effect of ASE in LPS-induced microglial BV2 cells. We first treated BV2 cells with three concentrations of ASE (10, 30, and 100 μg/mL) for 24 h and found that the treatment did not decrease the cell viability (Figure 6A). ASE was found to significantly inhibit the production of NO in LPS-stimulated BV2 microglial cells in a dose-dependent manner (Figure 6B). Specifically, the highest concentration of ASE (100 μg/mL) exhibited the most pronounced inhibitory effect, suggesting that ASE can effectively modulate the inflammatory response in microglial cells. Therefore, in accordance with the anti-inflammatory effects of the four major components in Yinchen, ASE extracted from Yinchen could also inhibit the neuroinflammation in vitro.

### 2.11. ASE Attenuates Neurological Functional Injury After tMCAO

**Figure 7:**
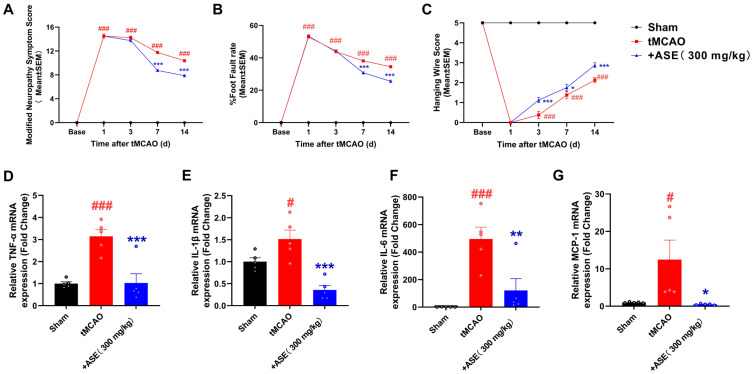
Effects of ASE on tMCAO rats. (**A**) ASE decreased the mNSS of the tMCAO rats. (**B**) ASE decreased the foot fault rate of the tMCAO rats. (**C**) ASE increased the hanging wire score of the tMCAO rats (*n* = 8). (**D**–**G**) Quantitative evaluation of TNF-α, IL-1β, IL-6, and MCP-1 levels in the ischemic brain (*n* = 5). Data are expressed as mean ± SEM. ^#^ *p* < 0.05 and ^###^ *p* < 0.001 vs. the Sham group; * *p* < 0.05, ** *p* < 0.01 and *** *p* < 0.001 vs. the tMCAO group.

To further investigate the therapeutic effect of ASE against IS in vivo, we established the tMCAO model in Wistar rats. The results showed that the modified neurological severity scores (mNSS) of the tMCAO rats were significantly higher than those of the sham-operated group. However, after treatment, the ASE-treated rats exhibited markedly lower scores than those of the tMCAO rats during the late stage of administration (Figure 7A). Compared with the sham group, the foot-fault rate of tMCAO rats on the wire grid was significantly elevated, while ASE reduced this rate in tMCAO rats (Figure 7B). In the hanging wire test, the scores of tMCAO rats were significantly lower than those of the sham group after reperfusion; following drug administration, the ASE-treated group showed a time-dependent increase, with scores significantly higher than those of the tMCAO group (Figure 7C). Therefore, ASE extracted from Yinchen demonstrated the neurological outcome-improving effect in rats post-stroke, suggesting that Yinchen might have a therapeutic effect against IS.

### 2.12. ASE Alleviates the Inflammation in the Ischemic Brain of tMCAO Rats

To further investigate the potential mechanism by which ASE acts on tMCAO rats, we measured the expression of pro-inflammatory cytokines (TNF-α, IL-1β, IL-6, and MCP-1) in the ischemic border zone (IBZ) of the brain. As shown in Figure 7D–G, the expression of TNF-α, IL-1β, IL-6, and MCP-1 was significantly higher in the ischemic brain of the tMCAO group compared with the sham group. After the administration of ASE, the levels of these pro-inflammatory cytokines were markedly decreased compared with the tMCAO group, which was consistent with ASE’s in vitro anti-neuroinflammatory results. Therefore, ASE could improve neurological function outcomes in tMCAO rats via inhibition of neuroinflammation.

In this study, network pharmacology predicted that 103 compounds of Yinchen had 198 intersecting targets with IS. Also, the top five Yinchen and IS intersecting core targets were TNF, STAT3, IL1B, AKT1, and SRC. Molecular docking results demonstrated that four major active compounds, including isochlorogenic acid C, isochlorogenic acid B, isochlorogenic acid A, and chlorogenic acid, identified by HPLC and MS in ASE, showed good interaction with all the above five core targets. Both the four compounds and ASE were observed to attenuate NO release and suppress the release of the inflammatory factors (TNF-α, IL-1β, IL-6, and MCP-1) in a dose-dependent manner in LPS-induced BV2 microglial cells, and ASE was further found to exert neuroprotective effects against I/R injury and inhibit the production of inflammatory factors in tMCAO rats. These findings suggest that Yinchen has potential as a candidate drug for IS treatment through its anti-inflammatory effects, and four major active compounds identified in Yinchen might constitute the material basis of Yinchen’s effect against IS.

## 3. Discussion

In the present study, we demonstrated that ASE, which is extracted from the TCM component Yinchen, improves neurological outcomes in tMCAO rats by mitigating neuroinflammation. Both ASE and its four characteristic constituents (isochlorogenic acid C, isochlorogenic acid B, isochlorogenic acid A, and chlorogenic acid) exhibited strong binding affinities for the five inflammatory and IS-associated targets (TNF, STAT3, IL1B, AKT1, and SRC) that we identified, and were further found to reduce the production of NO and the generation of pro-inflammatory factors (TNF-α, IL-1β, IL-6, and MCP-1) in LPS-induced BV2 microglial cells. While the traditional formula, Zhengan Xifeng Decoction, which contains Yinchen, has been used to treat IS [17], the specific role and underlying mechanism of Yinchen itself in IS therapy remained unclear. These findings confirmed our hypothesis that Yinchen possesses therapeutic potential for IS. Furthermore, we identified several of its constituents as promising anti-IS candidates, establishing a material basis for Yinchen’s efficacy, which is primarily mediated through its potent anti-inflammatory properties both in vivo and in vitro.

Inflammation plays a critical role in the pathogenesis of IS. In the progression of IS, inflammatory cytokines and chemokines are produced, exacerbating neuronal damage and neurovascular injury during stroke [20]. Therefore, combating neuroinflammation is a promising strategy for the treatment of IS. In order to pinpoint the potential key targets of Yinchen for IS treatment, we conducted PPI system analysis. Our results demonstrate that five core genes in Yinchen associated with inflammation and IS, including TNF, STAT3, IL1B, AKT1, and SRC, are significantly related to the treatment of IS. Among them, TNF is an inflammatory cytokine. When brain cells are damaged by ischemia, neurons, microglia, and vascular endothelial cells produce TNF-α. The higher the concentration of serum TNF-α, the larger the volume of cerebral infarction and the more severe the disease is. Therefore, TNF-α is one of the clinical test markers for nerve cell injury after ischemia [21]. As a chemokine, IL-1β can not only transmit information and regulate immune cells and inflammatory factors; it can also transduce the activation and proliferation of T and B lymphocytes. IL-1β promotes the release of neurotoxicity and aggravates brain nerve damage. At the same time, the expression level of inflammation in the body is up-regulated, which further aggravates post-ischemic injury. TNF-α and IL-1β, as the initiating substances in the inflammatory response chain, are involved in the whole process of stroke and can trigger an inflammatory cascade. After infarction, the content of both is higher than normal [22]. Our research also revealed that in the classic tMCAO stroke model, the levels of TNF-α and IL-1β, two of the five core target genes, increased significantly. Su’s study demonstrated that Yinchen and a Gancao Decoction significantly reduced the expression levels of IL-1β, IL-6, and TNF-α in the hepatocyte and revealed that hepatocyte necrosis caused by cholestatic liver injury can be alleviated by reducing the release of inflammatory cytokines [23]. Similarly, our research also found that ASE induced an improvement of neurological function and had anti-inflammatory effects in tMCAO rats. It also had the effect of inhibiting the release of pro-inflammatory factors, including TNF-α, IL-6, IL-1β, and MCP-1, and the downregulation of these factors was dose-dependent. Therefore, the effectiveness and accuracy of the five core targets were confirmed through network pharmacology analysis and the subsequent experimental study results, which demonstrated that Yinchen, when used as a single medicinal herb, has potential beneficial effects against IS by attenuating neuroinflammation via modulating the top five Yinchen and IS intersecting core targets. Still, the mechanism by which the top five core targets are regulated by Yinchen remains to be elucidated.

The constituents interacting with the top five Yinchen and IS intersecting core targets may be responsible for Yinchen’s effect against IS. Through HPLC-HRESIMS and HPLC-UV analyses, four active compounds, including isochlorogenic acid C, isochlorogenic acid B, isochlorogenic acid A, and chlorogenic acid, were identified in ASE, and all of them showed good scores in their ability to interact with all of the above five core inflammatory and IS-associated targets. As is consistent with ASE, these four compounds can also reduce the levels of NO and the pro-inflammatory factors (TNF-α, IL-1β, IL-6, and MCP-1). This further proves that it is specifically the interaction between TNF and IL1B among the five core targets that enables Yinchen’s anti-neuroinflammatory effect. Our results are consistent with those previously reported in the literature, demonstrating that these four compounds exert anti-inflammatory effects in various diseases [24,25,26,27,28]. A renal herb formula with several characteristic components, including isochlorogenic acid C, was found to protect against hyperuricemic nephropathy by inhibiting apoptosis and inflammation [24]. Also, isochlorogenic acid B was observed to alleviate Pb-induced inflammation in the brain, as indicated by decreasing TNF-α and IL-6 levels [25]. Moreover, isochlorogenic acid A was reported to improve liver fibrosis and inflammation by inhibiting toll-like receptor 4 (TLR4)/NF-κB pathways [26]. Although all four compounds have been reported to have anti-inflammatory activities, chlorogenic acid is the only one reported to be able to alleviate IS by inhibiting TLR4-mediated neuroinflammation [27]. Also, the effects of the other three major constituents in Yinchen against IS or their anti-neuroinflammatory role in IS have not been specifically studied yet. Chlorogenic acid was found to primarily restrain the synthesis and release of inflammatory mediators such as TNF-α, NO, cyclooxygenase-2 (COX-2), and PGE2 [28], which was consistent with our experimental results, which showed that it significantly inhibited NO production and reduced TNF-α levels in a dose-dependent manner in LPS-stimulated BV2 microglial cells. Therefore, our preliminary study indicated that the identified ingredients in both Yinchen and ASE exhibited anti-neuroinflammatory effects, and ASE was shown to improve neurological function in tMCAO rats. These findings suggest Yinchen’s therapeutic potential for IS, though further research is needed to elucidate its underlying mechanisms and the precise material basis of its effects against neuroinflammation in IS.

In addition to acting on the inflammatory pathways related to TNF-α and IL-1β among the five core targets, KEGG analysis results further demonstrated several signaling pathways related to the potential treatment of IS by Yinchen. The enriched KEGG pathways included the MAPK signaling pathway, the calcium signaling pathways, the cGMP-PKG signaling pathway, the inflammatory mediator for regulation of TRP channels, the NF-κB signaling pathway, etc. Studies have shown that reduced activation of the NF-κB and MAPK signaling pathways can result in decreased expression and activation of the NOD-like receptor family pyrin domain containing 1 (NLRP1) and NLRP3 inflammasomes, as well as increased expression of anti-apoptotic proteins Bcl-2 and Bcl-xL in primary cortical neurons and/or cerebral tissue under in vitro and in vivo ischemic conditions, thus reducing neuronal cell death and brain injury following IS [29]. SRC is one of the five core targets and is found to be strongly associated with the inflammatory process in IS [30]. The pretreatment of agomiR-203a-3p and agomiR-153-3p improved IS-induced neuronal apoptosis by inhibiting the SRC-dependent MAPK signaling pathway, and the inhibition of SRC expression could reduce the level of the expression of NLRP3 inflammasome-related factors [31]. In addition, the serine/threonine protein kinase known as AKT1 is involved in a variety of physiological and pathological processes, including cell differentiation, apoptosis, inflammation, and metabolism following ischemia [32,33]. Once activated, AKT1 triggers a series of signal cascade reactions, which can reduce the death of brain cells, promote the growth of neural cells and vascular endothelial cells, enhance the regeneration and repair of nerve tissue and vessels, and improve neural function after cerebral ischemia [34]. In addition, the involvement of the janus kinase 2 (JAK2)/STAT3 pathway is crucial in the pathological process underlying IS. The increased expression of JAK2/STAT3 observed in individuals suffering from acute IS may contribute to cellular inflammation [35]. Therefore, the role of other signaling pathways in the potential of Yinchen to treat IS is worth further investigation.

Though our study has verified the therapeutic potential of Yinchen against IS and elucidated its material basis through network pharmacology analysis and experimental study, it has certain limitations. Firstly, due to the inherent limitations of network pharmacology in comprehensively mapping bioactive entities, certain compounds and their corresponding target genes might be absent from public databases. Secondly, in this study, we established the classic tMCAO rat model of IS and preliminarily confirmed the neuroprotective effect of ASE and its inhibitory effects on pro-inflammatory cytokines in rat ischemic brain tissues. There is still a lack of evidence for its clinical application. Thirdly, though network pharmacology and our preliminary results have demonstrated the critical role of the identified inflammatory core targets and biological processes in enabling Yinchen’s effect against IS, the mechanisms for how Yinchen or its characteristic components specifically modulate these pathways were not investigated in this study. Our future research will still need to evaluate the potential therapeutic mechanisms of Yinchen against IS both in vivo and in vitro.

## 4. Materials and Methods

### 4.1. Preparation of the ASE

The aerial parts of *Artemisia scoparia* Waldst. et Kit. were harvested in July 2014 from Urumqi, Xinjiang Uygur Autonomous Region, China. An authenticated voucher specimen (No. 20141227) has been archived at the Xinjiang Institute of Materia Medica, with botanical verification conducted by Associate Professor Jiang He from the same institution. Ten kilograms of dried plant material underwent mechanical fragmentation followed by triple aqueous extraction (150 L of water per cycle, 1 h of reflux per extraction). After filtration and vacuum concentration to a relative density of 1.2–1.25 (60 °C measurement), the condensate underwent tenfold aqueous dilution. The diluted solution underwent 12 h adsorption pretreatment before chromatographic separation through a D101 macroporous resin column, using 70% ethanol as eluent. The collected fractions were concentrated under reduced pressure to achieve identical density parameters (1.2–1.25 at 60 °C), followed by vacuum desiccation at 60 °C to yield 74 g of ASE [16].

### 4.2. HPLC-UV (DAD) Analysis and UV Spectra of the Major Peaks of the ASE

A quality control analysis of ASE, isochlorogenic acid C, isochlorogenic acid B, isochlorogenic acid A, and chlorogenic acid was performed using HPLC-DAD. Analytical HPLC was performed on a Thermo Ultimate 3000 instrument (Thermo, Waltham, MA, USA) using a Nano Chrom 120 C18 column (250 × 4.6 mm, 5 μm) with a flow rate of 1.0 mL/min, and the gradient program of MeCN/0.1% H_3_PO_4_ in H_2_O was 10:90 (t = 0 min), 15:85 (t = 10 min), 20:80 (t = 30 min), 30:70 (t = 35 min), 35:65 (t = 50 min), 40:60 (t = 55 min), 55:45 (t = 60 min), 65:35 (t = 65 min), and 90:10 (t = 70 min).

### 4.3. High-Resolution Mass Measurements of Constituents in the ASE

HPLC-UV analyses were performed using a Dionex Ultimate 300 UPLC system (Thermo, Waltham, MA, USA) coupled with a Thermo QExactive Focus (Thermo, Waltham, MA, USA). HPLC separation was carried out on Nano Chrom 120 C18 column (250 × 4.6 mm, 5 μm) with a flow rate of 1.0 mL/min, and the gradient program of MeCN/0.1% formic acid in H_2_O was 10:90 (t = 0 min), 15:85 (t = 10 min), 20:80 (t = 30 min), 30:70 (t = 35 min), 35:65 (t = 50 min), 40:60 (t = 55 min), 55:45 (t = 60 min), 65:35 (t = 65 min), and 90:10 (t = 70 min). The ESI mass spectrometer was equipped with a binary pump, a DAD, a vacuum degasser, an autosampler, and a column heater–cooler (Thermo, Waltham, MA, USA). The MS conditions were set as follows: Spray voltage: 3.3 kV (+), 2.8 kV (−), sheath gas flow rate: 35 PSI, aux gas flow rate: 10Arb, capillary temperature: 32 °C, flow rate: 1.0 mL/min, split ratio: 1:2.

### 4.4. Screening of Effective Components and Prediction of Targets

The chemical constituents and 3D structural formulas of Yinchen were obtained by using TCMSP (https://tcmsp-e.com, accessed on 22 January 2024), TCM Database@Taiwan (http://tcm.cmu.edu.tw, accessed on 22 January 2024), and the Chinese Natural Products Chemical Component Database (https://www.pharmdata.ac.cn/, accessed on 22 January 2024). The results were combined, and the duplicate values were deleted. According to Lipinski’s Rules, that is, relative molecular mass <500, number of hydrogen bond donors <5, number of hydrogen bond acceptors <10, oil–water partition coefficient <5, and number of rotatable bonds ≤10, the chemical constituents of Yinchen were analyzed by Discovery studio 2019, and the ADMET descriptors module was used to evaluate the chemical constituents. The effective components with good water solubility and high intestinal bioavailability were selected for subsequent analysis. The structural formulas of the screened active ingredients were imported into the SwissTargetPrediction database (http://swisstargetprediction.ch/, accessed on 25 January 2024) for target prediction, and the UniProt database (https://www.uniprot.org/, accessed on 25 January 2024) was used to standardize the obtained targets.

### 4.5. Construction of Drug-Disease Intersecting Targets

‘Ischemic Stroke’ was used as the keyword to search and screen in the GeneCards (https://www.genecards.org/, accessed on 28 January 2024) and DisGeNET (https://www.disgenet.org/, accessed on 28 January 2024) disease–gene databases. The results were combined, the duplicate values were deleted, and the UniProt database was used to standardize the obtained targets. Venny 2.1.0 (https://bioinfogp.cnb.csic.es/tools/venny/, accessed on 30 January 2024) was used to obtain the intersection between the targets of active ingredients in Yinchen and the disease targets, and the intersection was visualized by a Venn diagram.

### 4.6. Network Construction of ‘Drug–Active Ingredient–Intersecting Target–Disease’

The obtained relevant data were imported into the software Cytoscape 3.10.1 to construct the ‘drug-effective component–intersecting target–disease’ network of Yinchen. The ‘nodes’ represented the components and targets, and the ‘edges’ represented the relationship between the two. The Network Analyzer analysis tool was used to analyze the characteristics of the network.

### 4.7. Construction of Protein–Protein Interaction Network

The intersecting targets of Yinchen and IS were imported into the STRING database (https://cn.string-db.org/, accessed on 2 February 2024) to create a visual PPI network. The data were imported into Cytoscape 3.10.1 software, and the network was topologically analyzed by the Network Analyzer. The selection of the top five core interaction targets was based on sorting the node degrees from largest to smallest.

### 4.8. GO and KEGG Pathway Enrichment Analysis

The Metascape database (https://metascape.org/gp/index.html, accessed on 27 August 2024) was used for GO function analysis and KEGG pathway enrichment analysis, and the intersection targets of Yinchen and IS were uploaded to obtain relevant data. The top ten analysis results were selected, and the corresponding data were imported into the Wei Sheng Xin (http://www.bioinformatics.com.cn/, accessed on 27 August 2024) online mapping tool for visualization. The GO functional analysis bar chart included three parts: biological process, cell component, and molecular function, as well as the bubble diagram of the KEGG enrichment analysis.

### 4.9. Molecular Docking

Molecular docking was performed on the first five core intersection targets and the effective components, which were identified by HPLC-MS of Yinchen. The 3D structure of the proteins corresponding to the top five core targets was downloaded from the protein database PDB (Protein Data Bank) (https://www.rcsb.org/, accessed on 1 June 2025) as a protein receptor library. The 3D structures corresponding to the four identified active ingredients of Yinchen were downloaded from the PubChem database (https://pubchem.ncbi.nlm.nih.gov/, accessed on 1 June 2025). The Prepare Protein tool in Discovery Studio 2019 was used to pretreat the protein by removing water and hydrogenation, and the active center was defined by the Define and Edit Binding Site tool. The location and size of the active center were referred to the position of the protein ligand obtained from the PDB database. The CDOCKER function was then used to perform molecular docking and calculate the -CDOCKER interaction energy. The higher the docking score, the stronger the binding ability of the small ligand molecule to the receptor protein was. The docking scores of the components and targets were statistically analyzed. The agonists or antagonists of the five targets were retrieved from the IUPHAR database (https://www.guidetopharmacology.org, accessed on 1 June 2025), and the docking scores were used to assess the potential activity of the metabolites in Yinchen. The following agonists and antagonists were used for molecular docking: TNF (antagonist: SPD-304); STAT3 (antagonist: S3I-201); IL1B (antagonist: Diacerein); AKT1 (agonist: SC79); and SRC (antagonist: KX2-391).

### 4.10. Drugs and Reagents

Isochlorogenic acid A, isochlorogenic acid B, isochlorogenic acid C, and dexamethasone were purchased from Shanghai Yuanye Bio-Technology Co., Ltd. (Shanghai, China); chlorogenic acid was purchased from Jiangsu Aikon Biopharmaceutical R&D Co., Ltd. (Heowns, Tianjin, China). The reagents were dissolved in DMSO (Aladdin, Beijing, China) and diluted in saline. Cell viability was evaluated using a CellTiter 96^®^ AQueous One Solution Cell Proliferation Assay (MTS assay, Promega, Madison, WI, USA). The Griess reagent assay kit was purchased from Applygen Gene Technology Co., Ltd. (Beijing, China). RT-qPCR was performed with specific primers and SYBR^®^ Premix Ex TaqTII (TakaRa Clontech, Dalian, China).

### 4.11. Cell Culture

The BV2 microglial cell line was obtained from the Cell Resource Center, Institute of Basic Medical Sciences, Chinese Academy of Medical Sciences & Peking Union Medical College (Beijing, China). The cells were maintained in Dulbecco’s modified Eagle medium (DMEM) (gibco, New York, NY, USA) supplemented with 10% fetal bovine serum (FBS) (PAN, Brisbane, Australia) at 37 °C in a humidified atmosphere of 5% CO_2_.

### 4.12. Cell Viability Assay

BV2 cells with a density of 2.5 × 10^4^ cells per well were seeded in 96-well plates, cultured at 37 °C overnight, and then incubated with ASE (10, 30, and 100 μg/mL) or compounds (1, 3, and 10 μM) for another 24 h. Subsequently, the cells were incubated with MTS reagent at 37 °C for 4 h, and then the absorbance was measured at 490 nm on a microplate reader [36]. Cell viability was calculated using the following formula: (OD_Experimental group_ − OD_Blank_)/(OD_Control group_ − OD_Blank_) × 100%.

### 4.13. NO Assay

BV2 cells were seeded in 96-well plates at a density of 2.5 × 10^4^ cells per well and incubated for 24 h. The cells were pretreated with compounds at the following stated concentrations (1, 3, and 10 μM) or ASE at the following specified concentrations (10, 30, and 100 μg/mL) for 2 h, followed by stimulation with LPS at 1 μg/mL for 24 h. After the incubation period, 50 μL of cell supernatant was collected, and NO levels were measured using a Griess reagent assay kit. The absorbance was then detected at 540 nm with a microplate reader. The nitrate concentration was calculated according to a standard curve generated from known concentrations of sodium nitrite [37].

### 4.14. Real-Time Quantitative Reverse Transcription RT-qPCR

Total RNA was extracted from the cells or the rat IBZ brain tissues using the trizol method and was reverse transcribed using the HiScript III All-in-One RT SuperMix Perfect for qPCR (Vazyme, Nanjing, China). A qPCR (2 × AceQ Universal SYBR qPCR Master Mix, Vazyme, Nanjing, China) was subsequently performed using the CFX96 Touch Real-Time PCR Detection System (Bio-Rad, Hercules, CA, USA). Reactions were performed following the manufacturer’s instructions. The primer sequences were based on previously published primer sequences [36]. The gene expression levels were quantified using the 2^−ΔΔCq^ method and normalized to the internal reference gene β-actin [36].

### 4.15. Animals

Male adult Wistar rats (weighing 280–320 g) obtained from Beijing Vital River Laboratory Animal Technology Co., Ltd. (Beijing, China) were used in this study. Animals were housed under standard conditions (temperature 24 ± 1 °C; humidity 50–60%; a 12 h light/dark cycle) and had free access to food and water. The Animal Ethics Committee of the Chinese Academy of Medical Sciences and Peking Union Medical College approved the animal protocols before the start of any protocol-specified procedures (IMM-S-25-0038).

### 4.16. tMCAO

Rats were fasted overnight and housed at 25 °C with free access to water. They were anesthetized with 4% isoflurane in 70% N_2_O/30% O_2_ and then maintained at 3% isoflurane. Rectal temperature was kept at 37 ± 0.5 °C using a feedback-controlled heating pad. After making a skin incision midway between the left orbit and the external auditory canal, the left common carotid artery, external carotid artery (ECA), and internal carotid artery (ICA) were exposed. A 0.38 mm diameter of silicone-coated monofilament suture was inserted into the ECA and advanced into the ICA until slight resistance was felt, thereby occluding the origin of the middle cerebral artery. After 1.5 h of occlusion, the filament was gently withdrawn to restore blood flow; successful reperfusion was confirmed by immediate hyperemic flushing of the ICA [38]. The incision was closed, and animals were allowed to recover in a warmed cage. Sham-operated rats (sham group) underwent an identical procedure except for the occlusion step.

### 4.17. ASE Administration

The rats were randomly assigned into 3 experimental groups of 8 rats each, as follows: sham group, tMCAO group, and ASE + tMCAO group (300 mg/kg) were given an intragastric administration (i.g.) of ASE as an aqueous suspension in 0.5% sodium carboxymethyl cellulose (CMC-Na) starting 24 h after tMCAO and repeated daily for a total of 14 days, and the sham and tMCAO groups were given an equivalent volume of 0.5% CMC-Na.

### 4.18. Behavioral Tests

Three behavioral tests were used to assess neurological deficits after tMCAO: mNSS, the foot-fault test, and the hanging wire test. The mNSS is an integrated assessment that includes tests of motor function, sensory function, reflexes, and balance. It was evaluated using a scale from 0 to 18, with 0 representing normal function and 18 indicating the most severe impairment [39]. A modified foot-fault test was employed to measure forelimb placement dysfunction. The total number of steps (movement of each forelimb) that the rat used to cross the grid and the total number of foot faults for the left forelimb were recorded [40]. The hanging wire test was used to evaluate both limb strength and balance after stroke [41]. These tests were evaluated on days 1, 3, 7, and 14 after the performance of tMCAO. All surviving animals were euthanized 14 days after tMCAO.

### 4.19. Statistical Analysis

Data are presented as the mean ± SEM. Statistical analyses were performed using one-way analysis of variance (ANOVA) followed by Dunnett’s multiple-comparisons test or two-way ANOVA followed by Tukey’s multiple-comparisons test. The number of samples per cell group and the number of animals per group are specified in the figure legends. GraphPad Prism 9.0 software (GraphPad Software Inc., La Jolla, CA, USA) was used for all statistical analyses. A *p*-value < 0.05 was established as the criterion for statistical significance. Post hoc power analysis for the statistical test power of this study was conducted using G*Power 3.1.9.7 software (Heinrich Heine University, Düsseldorf, Germany). The analysis was based on the actual sample size, the predefined significance level (α = 0.05), and the effect size observed in the experiment. The test power (1−β) for all intergroup comparisons was 1.0.

## 5. Conclusions

In this study, network pharmacology and molecular docking technology were used to systematically analyze the action of Yinchen in the treatment of IS, revealing a process characterized by multi-component, multi-target, and multi-pathway action. Among Yinchen and IS’s intersecting targets, TNF, STAT3, IL1B, AKT1, and SRC were the top five core targets. Through HPLC-HRESIMS and HPLC-UV analyses, four compounds, including isochlorogenic acid C, isochlorogenic acid B, isochlorogenic acid A, and chlorogenic acid, were identified in ASE, which was extracted from Yinchen. These four major representative compounds showed good interaction with all of the above five core targets through molecular docking. Also, they were found to reduce the production of NO as well as the generation of inflammatory factors (TNF-α, IL-1β, IL-6, and MCP-1) in LPS-induced BV2 microglial cells dose-dependently. ASE also demonstrated anti-neuroinflammatory activity in an in vitro experiment. In vivo, ASE showed neurological functional injury attenuation and neuroinflammation alleviation effects in tMCAO rats. In conclusion, our results suggest that Yinchen has the potential to be a candidate drug for IS treatment through its anti-inflammatory effects.

Considering the limited treatment window and the post-stroke inflammatory cascade, ASE and other active components could be explored as an adjunctive therapy to current standards of care. Its role in mitigating secondary neuronal damage through neuroinflammation inhibition may help improve long-term neurological outcomes in stroke patients. This study provides a scientific foundation for the modern application of the traditional herb Yinchen, suggesting its potential utility beyond hepatobiliary disorders and bringing it into the realm of cerebrovascular diseases.

## Figures and Tables

**Table 1:** HPLC-HRESIMS data analyses of major constituents of ASE.

No.	Name	Formula	t_R_ (min)	(+)-HRESIMS *m/z*		(−)-HRESIMS *m/z*	
				Found	Calculated	Found	Calculated
1	Chlorogenic acid	C_16_H_18_O_9_	10.62	355.10211 [M + H]^+^	355.1029	353.08220 [M − H]^−^	353.0873
2	Isochlorogenic acid B	C_25_H_24_O_12_	30.61	517.13403 [M + H]^+^	517.1346	515.11975 [M − H]^−^	515.1190
3	Isochlorogenic acid A	C_25_H_24_O_12_	32.46	517.13409 [M + H]^+^ 499.12347 [M + H − H_2_O]^+^	517.1346	515.11975 [M − H]^−^	515.1190
4	Isochlorogenic acid C	C_25_H_24_O_12_	35.94	517.13391 [M + H]^+^	517.1346	515.11987 [M − H]^−^	515.1190

**Table 2:** The CDOCKER scores of the four compounds in Yinchen that interacted with the five core targets.

Compound	TNF	STAT3	IL1B	AKT1	SRC	Mean
Isochlorogenic acid C	68.13	53.47	80.03	73.38	50.54	65.11
Isochlorogenic acid B	70.61	48.85	62.73	77.81	52.06	62.41
Isochlorogenic acid A	67.5	53.72	76.29	67.4	43.13	61.61
Chlorogenic acid	58.39	34.88	57.21	52.46	54.05	51.40
Agonist/Antagonist	60.52	42.6	26.08	46.78	52.93	45.78

## Data Availability

The original contributions presented in this study are included in the article/Supplementary Material. Further inquiries can be directed to the corresponding author.

## Supplementary Materials

The following supporting information can be downloaded at https://www.mdpi.com/article/10.3390/ph18121852/s1. Table S1. The UV spectra, molecular formula, and structures of the major peaks of ASE detected by online HPLC-UV (DAD) and HPLC-HRESIMS. Figure S1. HPLC-UV (DAD) profiles of the ASE monitored at 210, 254, and 330 nm. Figure S2. HPLC-UV (DAD) profile and the UV spectrum of the marker compound chlorogenic acid (CGA) monitored at 330 nm. Figure S3. HPLC-UV (DAD) profile and the UV spectrum of the marker compound isochlorogenic acid B (ICGA-B, ICGB) monitored at 330 nm. Figure S4. HPLC-UV (DAD) profile and the UV spectrum of the marker compound isochlorogenic acid A (ICGA-A, ICGA) monitored at 330 nm. Figure S5. HPLC-UV (DAD) profile and the UV spectrum of the marker compound isochlorogenic acid C (ICGA-C, ICGC) monitored at 330 nm.

## Abbreviations

The following abbreviations are used in this manuscript:

ANOVA	analysis of variance
ASE	*Artemisia scoparia* extract
BBB	blood–brain barrier
cGMP-PKG	cyclic guanosine monophosphate-protein kinase G
CMC-Na	sodium carboxylmethyl cellulose
CGA	Chlorogenic acid
COX-2	cyclooxygenase-2
DAD	Diode array detector
DAMPs	damage-associated molecular patterns
DMEM	Dulbecco’s modified Eagle’s medium
ECA	external carotid artery
ET-1	endothelin-1
FBS	fetal bovine serum
GO	Gene Ontology
HREIMS	High Resolution Electrospray Ionization Mass Spectrometry
HPLC	high-performance liquid chromatography
IBZ	ischemic border zone
ICGA-A	Isochlorogenic acid A
ICGA-B	Isochlorogenic acid B
ICGA-C	Isochlorogenic acid C
ICA	internal carotid artery
IL-1β	interleukin-1β
IL-6	interleukin-6
IS	Ischemic stroke
JAK2	janus kinase 2
KEGG	Kyoto Encyclopedia of Genes and Genomes
LPS	Lipopolysaccharide
MAPK	mitogen-activated protein kinase
MCP-1	monocyte chemotactic protein-1
MMP-2	matrix metalloproteinase-2
mNSS	Modified Neurological Severity Score
NF-κB	nuclear factor kappa B
NLRP	NOD-like receptor family pyrin domain
NO	nitric oxide
NSS	neurological severity score
PDB	Protein Data Bank
PGE2	prostaglandin E2
PPI	Protein–protein interaction
qRT-PCR	Quantitative real-time PCR
RMSD	root mean square deviation
ROS	reactive oxygen species
r-tPA	recombinant tissue plasminogen activator
SRC	Src proto-oncogene, non-receptor tyrosine kinase
STAT3	signal transducer and activator of transcription 3
TCM	Traditional Chinese medicine
TLR4	toll-like receptor 4
tMCAO	Transient Middle Cerebral Artery Occlusion
TNF-α	tumor necrosis factor-α
TRP	transient receptor potential
