# Supplementary material for: Network Pharmacology Analysis and Experimental Study of Yinchen Against Neuroinflammation in Ischemic Stroke
Source: Pharmaceuticals (Basel). 2025 Dec 4;18(12):1852. doi: 10.3390/ph18121852 (PMC12735668; doi:10.3390/ph18121852)
Supplement: Supplementary file 1 [file pharmaceuticals-18-01852-s001.zip › pharmaceuticals-3894493-supplementary.pdf]

Supplementary Materials

Table S1. The UV spectra, molecular formula, and structures of the major peaks of ASE detected by on-line HPLC-UV (DAD) and HPLC-HRESIMS.

Peak t_R (min) [a]	UV spectra	Molecular formula [a]	Marker compounds
10.270	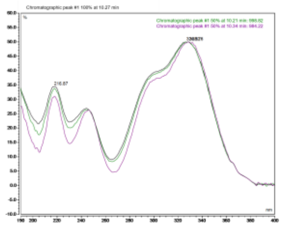	$C_{16}H_{18}O_9$	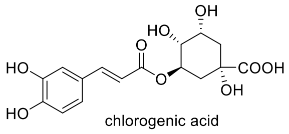 chlorogenic acid
17.477	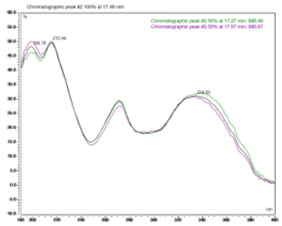	$C_{26}H_{28}O_{14}$	-[b]
23.813	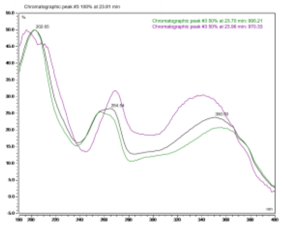	$C_{21}H_{20}O_{10}$	-[b]
30.837	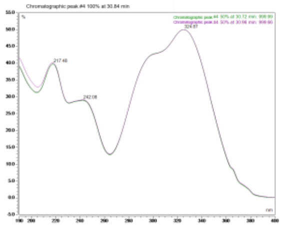	$C_{25}H_{24}O_{12}$	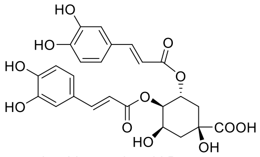 isochlorogenic acid B
32.683	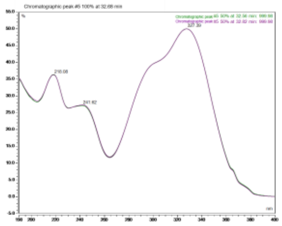	$C_{25}H_{24}O_{12}$	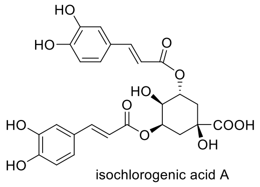 isochlorogenic acid A
36.377	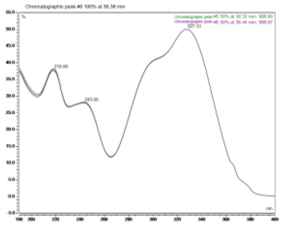	$C_{25}H_{24}O_{12}$	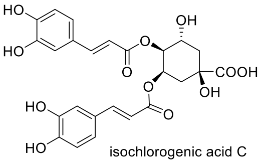 isochlorogenic acid C

42.243	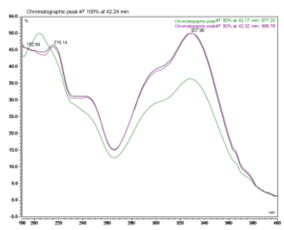	$C_{25}H_{22}O_{11}$	-[b]
47.703	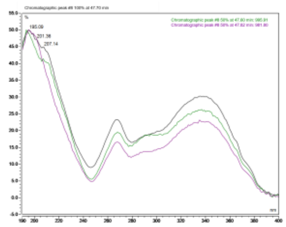	$C_{16}H_{12}O_{6}$	-[b]
48.477	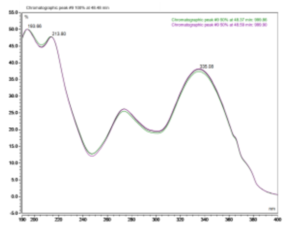	$C_{15}H_{20}O_{3}$	-[b]

[a] Molecular formula determined by the HRESIMS data (see Table 1);

[b] Their structures were not determined.

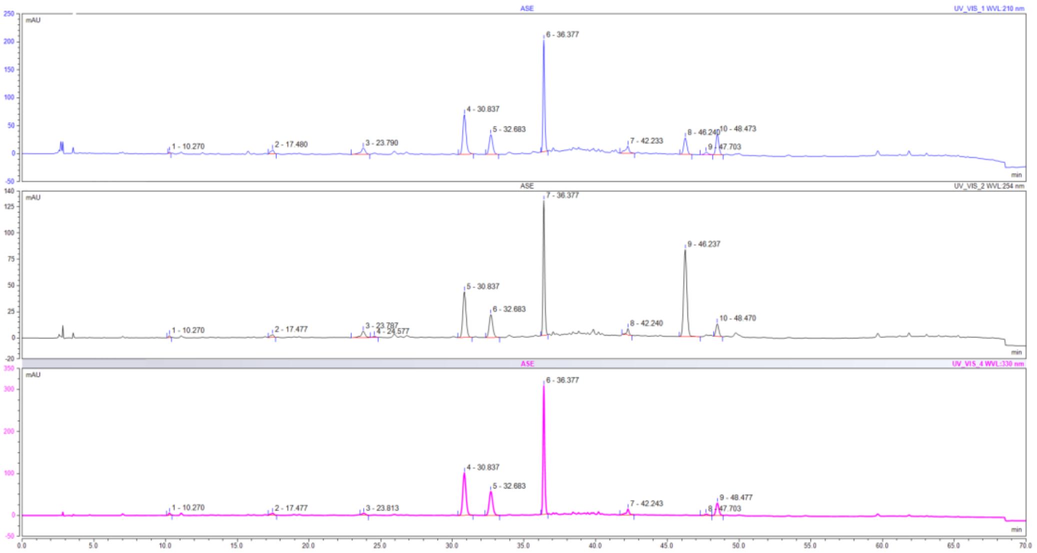

Figure S1. HPLC-UV (DAD) profiles of the ASE monitored at 210, 254 and 330 nm.

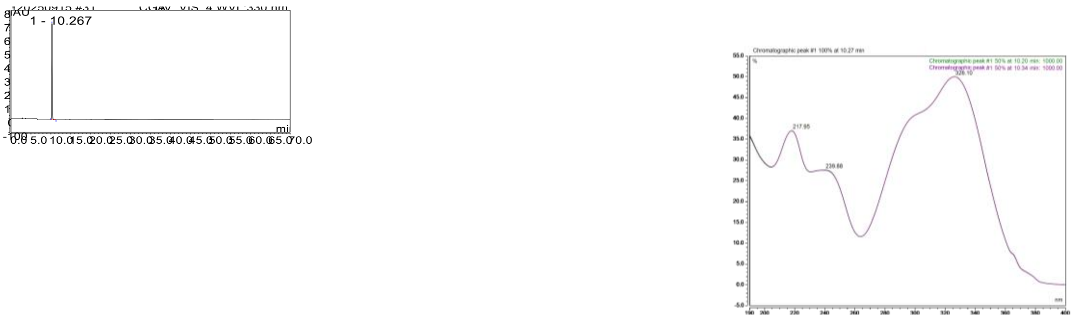

Figure S2. HPLC-UV (DAD) profile and the UV spectrum of the marker compound chlorogenic acid (CGA) monitored at 330 nm.

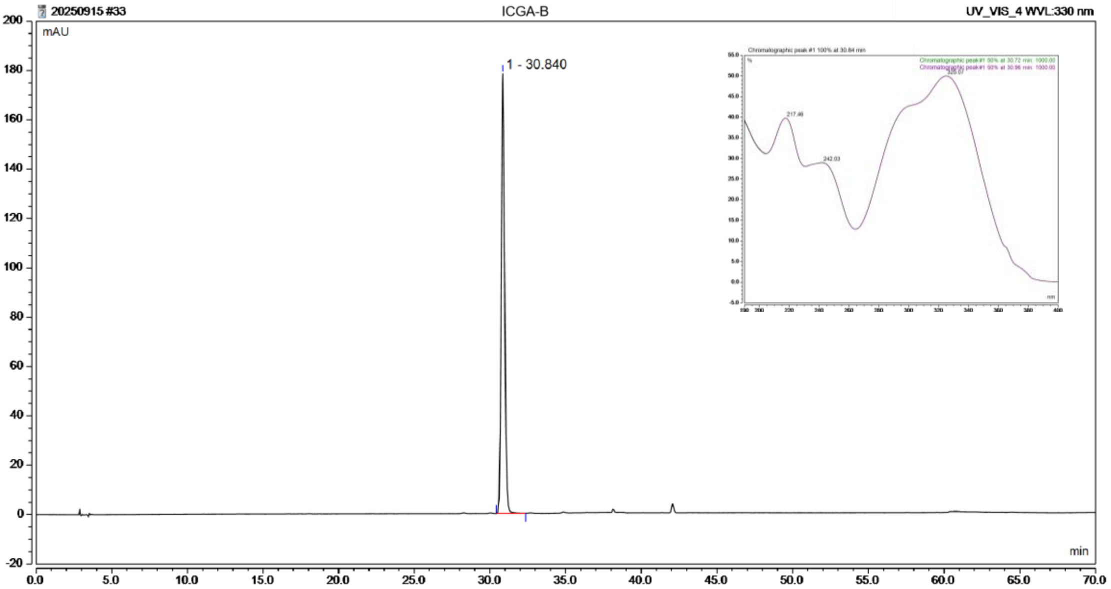

Figure S3. HPLC-UV (DAD) profile and the UV spectrum of the marker compound isochlorogenic acid B (ICGA-B) monitored at 330 nm.

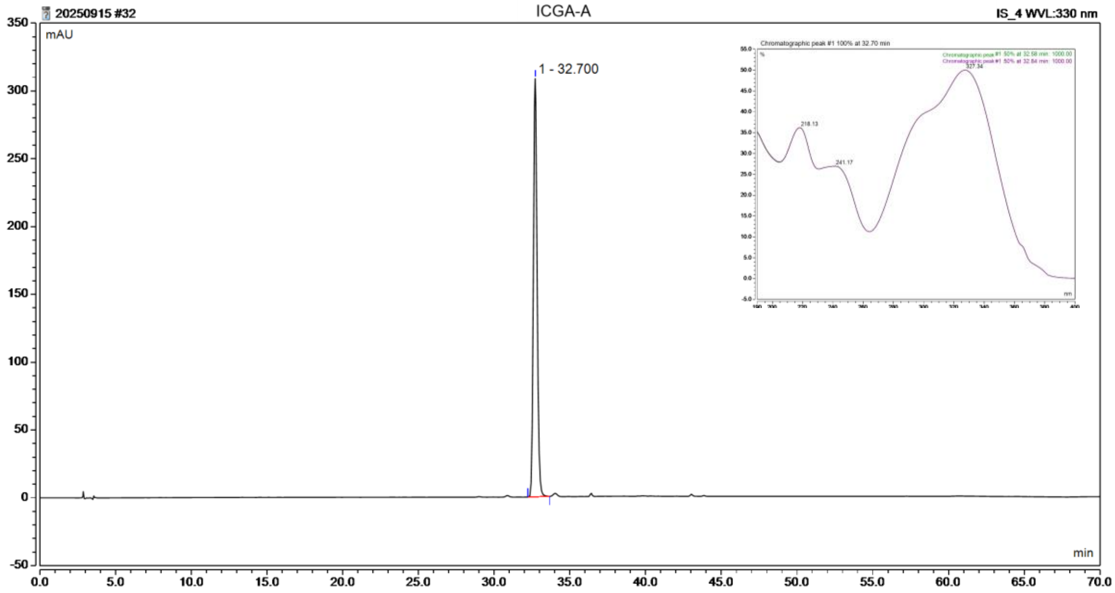

Figure S4. HPLC-UV (DAD) profile and the UV spectrum of the marker compound isochlorogenic acid A (ICGA-A) monitored at 330 nm.

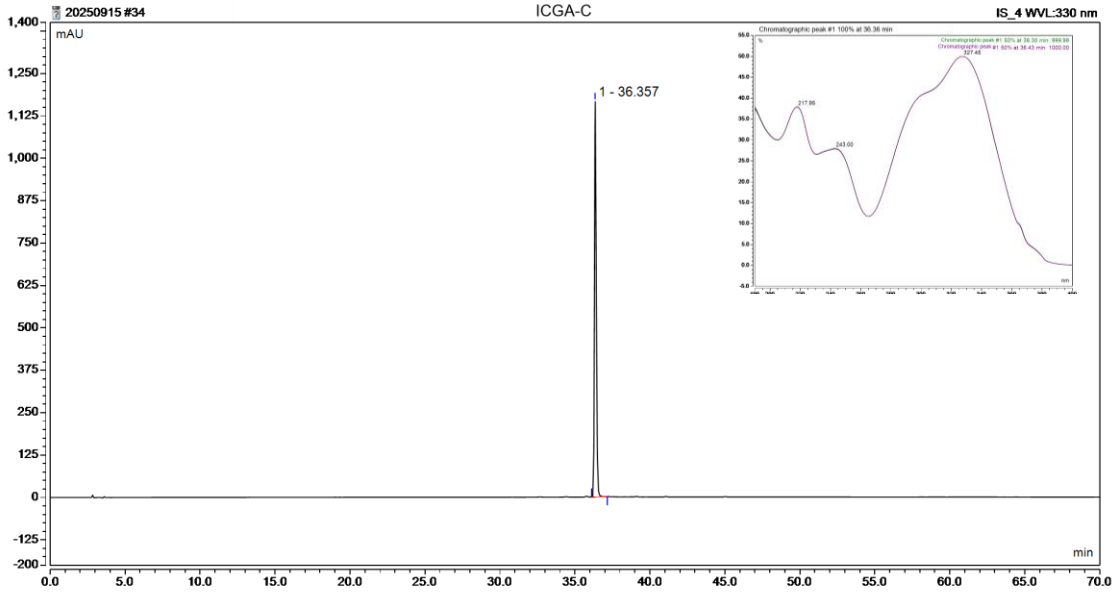

Figure S5. HPLC-UV (DAD) profile and the UV spectrum of the marker compound isochlorogenic acid C (ICGA-C) monitored at 330 nm.
